# Is adaptive therapy natural?

**DOI:** 10.1371/journal.pbio.2007066

**Published:** 2018-10-02

**Authors:** Frédéric Thomas, Emmanuel Donnadieu, Guillaume M. Charriere, Camille Jacqueline, Aurélie Tasiemski, Pascal Pujol, François Renaud, Benjamin Roche, Rodrigo Hamede, Joel Brown, Robert Gatenby, Beata Ujvari

**Affiliations:** 1 Centre de Recherches Ecologiques et Evolutives sur le Cancer/Maladies Infectieuses et Vecteurs: Ecologie, Génétique, Evolution, et Contrôle, CNRS, Université de Montpellier, Montpellier, France; 2 Inserm, Unité 1016, Institut Cochin, Paris, France; 3 Cnrs, Unité Mixte de Recherche 8104, Paris, France; 4 Université Paris Descartes, Sorbonne Paris Cité, Paris, France; 5 Interactions Host Pathogen Environment, University of Montpellier, Centre National de la Recherche Scientifique, Institut français de recherche pour l’exploitation de la mer, University of Perpignan Via Domitia, Montpellier, France; 6 Université de Lille-sciences et technologies, UMR 8198 Evo-Eco-Paleo, Villeneuve d’Ascq/CNRS/INSERM/CHU Lille, Institut Pasteur de Lille, U1019-Unité Mixte de Recherche 8204, Lille, France; 7 Service de génétique médicale et chromosomique, Unité d’oncogénétique, centre hospitalier régional et universitaire de Montpellier, Hôpital Arnaud de Villeneuve, Montpellier, France; 8 Unité mixte internationale de Modélisation Mathématique et Informatique des Systèmes Complexes, Sorbonne Université, BondyCedex, France; 9 Departamento de Etología, Fauna Silvestre y Animales de Laboratorio, Facultad de Medicina Veterinaria y Zootecnia, Universidad Nacional Autónoma de México, Ciudad de México, México; 10 School of Natural Sciences, University of Tasmania, Hobart, Tasmania, Australia; 11 Centre for Integrative Ecology, School of Life and Environmental Sciences, Deakin University, Waurn Ponds, Victoria, Australia; 12 Department of Radiology, H. Lee Moffitt Cancer Center & Research Institute, Tampa, Florida, United States of America; Georgia Tech, United States of America

## Abstract

Research suggests that progression-free survival can be prolonged by integrating evolutionary principles into clinical cancer treatment protocols. The goal is to prevent or slow the proliferation of resistant malignant cell populations. The logic behind this therapy relies on ecological and evolutionary processes. These same processes would be available to natural selection in decreasing the probability of an organism’s death due to cancer. We propose that organisms’ anticancer adaptions include not only ones for preventing cancer but also ones for directing and retarding the evolution of life-threatening cancer cells. We term this last strategy natural adaptive therapy (NAT). The body’s NAT might include a lower than otherwise possible immune response. A restrained immune response might forego maximum short-term kill rates. Restraint would forestall immune-resistant cancer cells and produce long-term durable control of the cancer population. Here, we define, develop, and explore the possibility of NAT. The discovery of NAT mechanisms could identify new strategies in tumor prevention and treatments. Furthermore, we discuss the potential risks of immunotherapies that force the immune system to ramp up the short-term kill rates of malignant cancer cells in a manner that undermines the body’s NAT and accelerates the evolution of immune resistance.

Cancer therapies, even when initially very effective, only rarely cure disseminated cancers. Intrinsic or acquired resistance by the cancer cells to treatment lead to relapse, progression, and death [1]. We propose that the emergence of resistant cancer cells requires two steps: first, the cells must deploy the necessary molecular machinery to overcome the toxic effects of the treatment. Second, the resistant cells must be sufficiently proliferative to repopulate the tumor. These steps must be deeply related to the cost of resistance. The resources needed to develop a resistant phenotype likely reduce fitness in the absence of therapy. Thus, wittingly or unwittingly, therapies govern the survivorship and proliferation of different cancer cell phenotypes within their tumor ecosystem.

Understanding the molecular basis of cancer drug resistance is a promising way to develop future treatments that could potentially circumvent or eliminate the problem of resistance [2–4]. Alternatively, strategies like adaptive therapy [5,6] focus on exploiting ecological (changes in the tumor size) and evolutionary dynamics (changes in the frequency of different cancer cell phenotypes) to delay or prevent the proliferation of resistant phenotypes (e.g., [7]). A critical issue arises when considering cancer therapy as an evolutionary process. The “brute force” therapy, aimed at killing the maximum number of malignant cells, can actually accelerate the evolution and proliferation of resistant cells. High-dose therapies allow resistant cancer cells to win twice. First, they are resistant. Second, they are free of competition from sensitive cancer cells. This leads to a proposed evolution-based strategy that enforces a stable tumor burden by permitting the persistence of a significant population of chemo-sensitive cells. In so doing, chemo-sensitive cells can outcompete chemo-resistant subpopulations, hence limiting their expansion. Since acquisition of chemo resistance generally requires significant investment of resources, cancer cells are subject to an evolutionary trade-off (due to the “cost” of phenotypic resistance) between resistance and proliferation [8,9].

Adaptive therapy was developed through mathematical models and computer simulations. In preclinical mouse studies and in a clinical trial on castrate-resistant metastatic prostate cancer, adaptive therapy delays or even prevents cancer progression as compared to traditional therapies, particularly those involving maximum tolerable dose [5]. Despite these successes, extensive further investigation is needed to define and understand evolutionarily optimal cancer treatment strategies [8,10].

The premise behind adaptive therapy is simple but relies on understanding the ecology and evolution of the cancer cell communities and their diversity of phenotypes. When “treatment to kill” is not possible for the metastatic disease, the goal should be to “treat to contain” in a manner that keeps the tumor burden below the level that threatens loss of life or even quality of life. In adaptive therapy, the treatment is used sparingly and in a temporally dynamic fashion (Fig 1A). The onset of therapy both reduces the tumor burden, drives down the population size of sensitive cells, and releases resistant cells from competition. Prior to driving the sensitive cells to near extinction, therapy is then withdrawn to permit their recovery. In the absence of therapy, the recovery of sensitive cells now has a competitive advantage over the resistant ones, thus acting to suppress them. Upon the recovery of the sensitive cell population, therapy is resumed, and the cycle repeats itself. The keys to the success of adaptive therapy in controlling (though not eliminating) the cancer cells are 1) the competitive advantage of sensitive cells relative to resistant ones in the absence of therapy, 2) the sparing use of therapy below that which would achieve maximum short-term kill rates, and 3) the strategic timing of therapy in response to overall tumor sizes and the frequencies of sensitive and resistant cell phenotypes.

**Fig 1:**
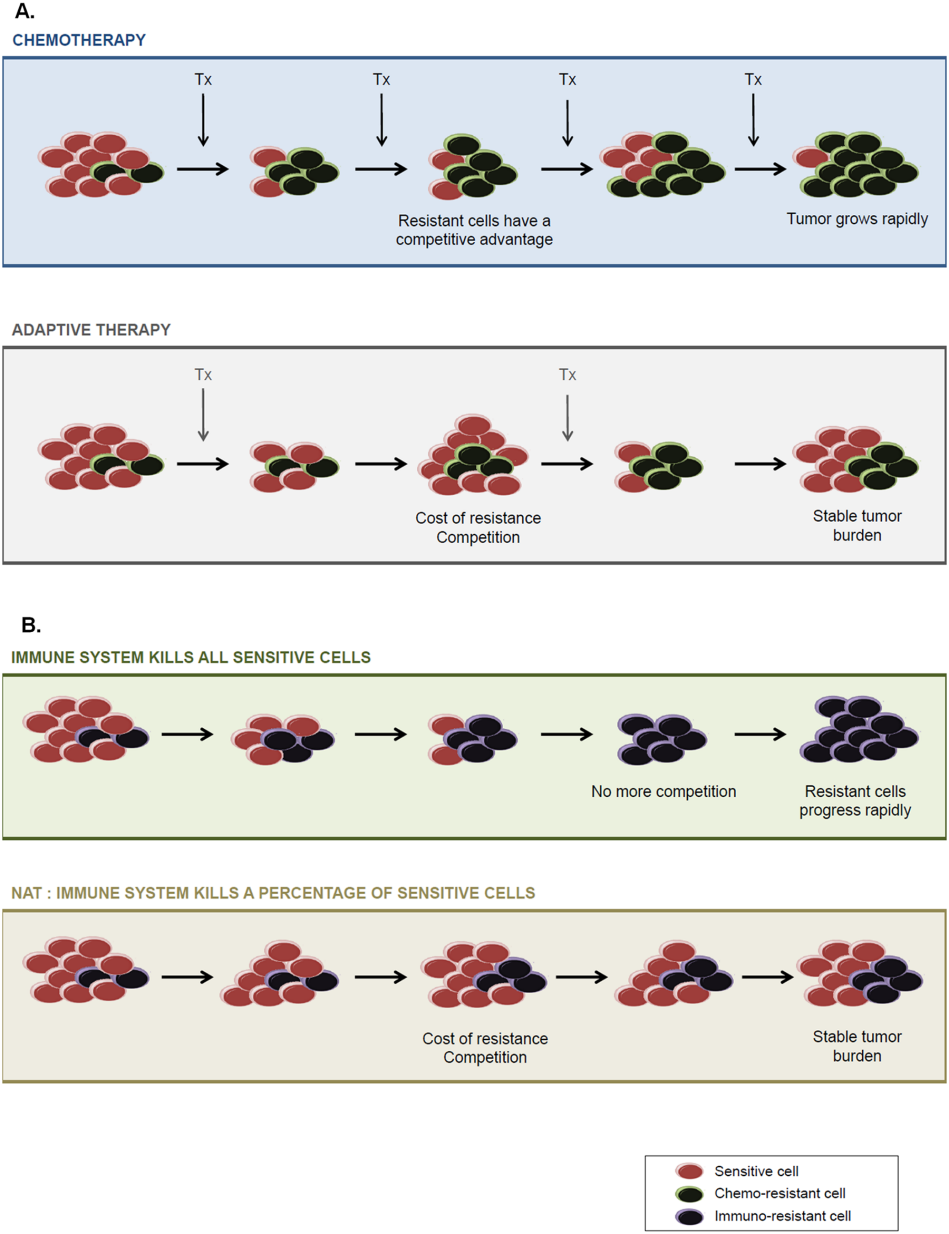
(A) Common strategies employed in chemotherapy and in adaptive therapy to deal with the proliferation of malignant cells. In adaptive therapy, treatment is used sparingly and in a temporally dynamic fashion, and this both increases the competitive advantage of chemo-sensitive cells and maintains a stable tumor burden. B) In a similar manner, when the immune system kills only some sensitive/visible tumor cells, it allows for maintenance of a stable tumor burden because this population competes with cells that are resistant to immune attack.

Here, we propose that the strategies employed in adaptive therapy may also be employed by multicellular organisms to deal with the inevitable development of malignant cells during growth and maintenance of normal tissue. Multicell organisms by way of natural selection over many generations may have evolved forms of natural adaptive therapy (NAT). Part of the organism’s anticancer adaptations may include containing rather than just eliminating or preventing cancers (Fig 1B). Such NAT could, for example, account for autopsy studies showing that small cancers are commonly present in people and animals who have died from noncancer causes. Such observations have led to a hypothesis that cancer emerges frequently, but host suppressive mechanisms, such as the immune system, successfully limit their proliferation in a manner that does not adversely affect host fitness. We also suggest explanations for some of the paradoxical relationships that seem to occur between the immune system and malignant cell populations. Why, for example, does the immune system appear to promote tumor growth under some conditions? [11,12]

It is widely accepted that the immune system is integral to tumor suppression. However, immunotherapies to increase that response may be counterproductive [13]. If our NAT is operating to contain the cancer cells and restrain their evolution of immune resistance, then forcing the immune system to increase kill rates may actually undermine this balance, quicken the evolution of resistant cancer cells, and reduce the time to cancer progression, as the resistant cancer cells are now free to proliferate independent of the immune system. By emphasizing short-term gains from the immune system, the host’s immune system may lose effectiveness and relevancy in the long term.

## Why are we not better at preventing and eliminating cancer? Current evolutionary hypotheses

Since the dawn of multicellularity (more than half a billion years ago), multicellular organisms have evolved cancer suppression mechanisms (e.g., apoptosis, effective DNA repair, DNA surveillance, epigenetic modifications, limited number of cell divisions, telomere shortening, tissue architecture, and immune surveillance) [14]. Given the billions, even trillions of cells present in the body of numerous multicellular species, these natural defenses against cancer are remarkably effective [15,16]. They are, however, not perfect, as illustrated by the significant prevalence of cancers worldwide in both humans [17] and animals [18], as well as the extremely high prevalence of oncogenic processes in general [19–21]. The premature (i.e., before the end of the reproductive period) death of people from cancer is also indirect evidence that at least certain malignant cells have or evolve the ability to circumvent all of our defenses. Several hypotheses have been proposed by evolutionary biologists to explain this persistent vulnerability. For example, it has been argued (as for diseases in general, see [22,23]), that natural selection has mostly favored the evolution of protective mechanisms before or during reproductive ages (i.e., selection intensity declines with age [24]). Accordingly, age is also one of the strongest predictors of cancer and risk of death from cancer [25,26]. In addition, even during the reproductive period, the costs of adaptations against cancer might exceed the benefits [27]. Selection may in turn have favored “low-cost” mechanisms that do not involve complete tumor elimination. Anticancer adaptations may be costly (in terms of survivorship and fecundity) and subject to diminishing returns. For instance, because of the risk of inducing autoimmunity, immune tolerance to cancer might be favored over more aggressive activation of the immune system [28]. Suppression of malignant progression may also lead to trade-offs that could inhibit other essential functions such as cell proliferation for the maintenance (e.g., wound healing) [15] of normal tissue. The p53 protein has a well-known cancer-suppressive function but also leads to reduced tissue renewal and repair, stem cell deletion, and organismal aging [29]. Thomas and colleagues [30] recently proposed an organ-centered approach for defining the evolutionary trade-offs that govern host responses to cancers. Organ-specific protection may not be maximal or equal across all organs. Rather, each organ may exhibit anticancer adaptations commensurate with the consequences of that organ acquiring cancer, and the trade-offs associated with organ-specific versus whole organism mechanisms of tumor suppression. Hence, cancer suppression and cancer incidence should be more prominent in some organs than in others. Similarly, based on which fitness-limiting factor, in addition to cancer predominates (chronic somatic, cardiovascular, and/or infectious diseases, predation, and/or adverse environmental conditions like famine, drought, accidents, disturbances), different predictions can be made on how natural selection should adjust the extent of cancer suppression. Through time, environmental changes may also create an evolutionary mismatch between historical adaptations of the immune system and contemporary lifestyles, as, for instance, observed with childhood acute lymphoblastic leukaemia [31]. Here, we explore the hypothesis that evolutionary principles used in adaptive therapy may also be deployed in NAT within the context of the trade-offs governing host tumor suppression.

## Why and when should NAT work?

**Fig 2:**
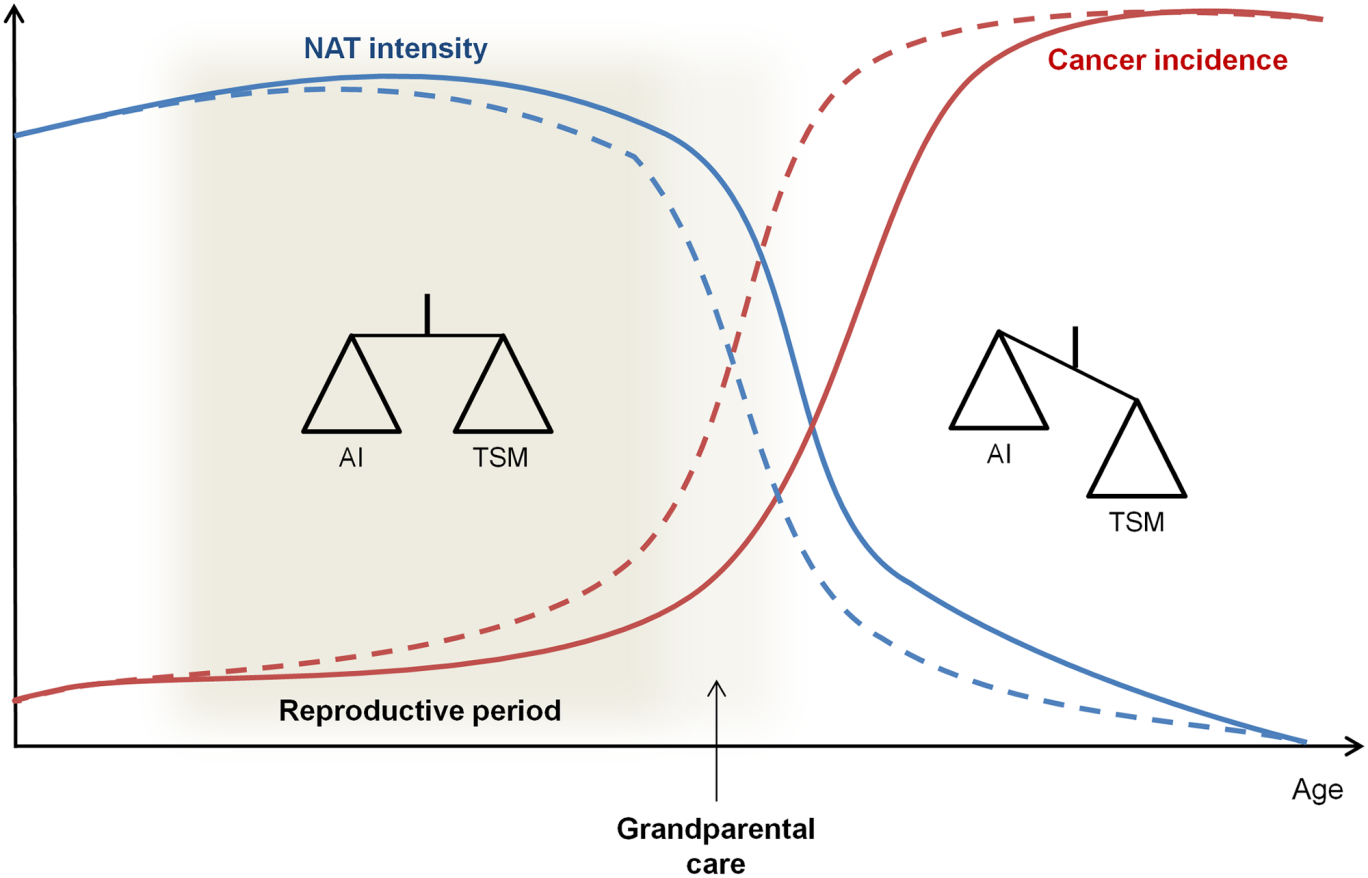
Evolution of the trade-off between TSMs and the risk of AI with age and reproductive status. During the reproductive period and before, NAT could result in a compromise between tumor suppression and the potential hazards to normal tissue to maintain cancer incidence at a low rate. This compromise could be extended in species in which there is grandparental care. After this reproductive period, NAT will decrease because tumor suppression costs will exceed the benefits, and tumor incidences will increase. Dashed lines are used for species without grandparental care and solid lines for species with grandparental care. AI, autoimmunity; NAT, natural adaptive therapy; TSM, tumor-suppressive mechanism.

The ultimate objective of adaptive therapy in cancer patients is to enhance their survival and quality of life [5]. Conversely, NAT, if it exists as an adaptation, will be evolutionarily optimized (as are all adaptations) to maximize fitness, given the circumstances. NAT is thus expected to have been shaped by selection that balances the benefits of tumor suppression against the potential hazards to normal tissue before and during the reproductive period and perhaps in species in which grandparental care affects survival (i.e., inclusive fitness, see [32]) (Fig 2). While optimal tumor control would rapidly eliminate any developing cancer population, this might have a significant cost in, for example, causing autoimmunity or limiting wound healing. Excessive immune responses against malignant cells could also contain the risk of preventing blastocysts from embedding in the endometrium and would therefore have detrimental fitness consequences. NAT would represent a compromise between these risks and benefits by allowing a sufficient host response to maintain stable small tumor burdens while minimizing the risk of damage to normal tissue and/or other fitness parameters. In particular, the immune system may permit the survival of some slowly growing tumor cells and allow this population, via intratumoral evolutionary dynamics, to suppress proliferation of cells that are more malignant and resistant to immune attack. We anticipate that the evolutionary compromises that govern NAT will vary between individuals within a population and probably between organs within each host. As with all host defenses, we propose that NAT will decrease with the end of the reproductive period.

Thus, while NAT is generally effective, cancers clearly develop in some individuals prior to the usual age of reproduction. In the optimization dynamics of evolution, this appears to be an “acceptable” compromise between trade-offs governing tumor suppression and the costs to normal tissue and whole organism function. Thus, cancer cells, through accumulating genetic and epigenetic events, evolve strategies to evade immune destruction [33].

NAT would have some clear advantages over those anticancer adaptations that aim to eliminate all aberrant cells or malignant populations of cells. Maintaining surveillance for potentially abnormal cells is likely metabolically costly. In addition to this ever-present fixed cost, there is likely the additional cost of eliminating cells that would not have advanced to malignancy or would have done so postreproductively or after death from other causes. Escalating an immune response to eliminate small populations of malignant cells, while eliminating potentially life-threatening cancer progression, has the same disadvantages as maximum tolerable dose therapies. The short-term gains become squandered on the rapid evolution of resistant cancer cells, and/or the therapy itself becomes life threatening. In maximizing fitness, natural selection integrates survival and fecundity across the distribution of expected lifetimes of the individuals comprising the evolving population. Hence, there is no reason to expect that natural selection will necessarily favor overly costly or risky “swift and sure” cancer suppression mechanisms. Rather, like adaptive therapy, natural selection would favor NAT if it significantly reduced costs, kept cancer mortality to a low enough level, and kept malignant populations of cells contained long enough to permit senescence or death from other causes. Finally, NAT has the advantage of being self-perpetuating. By slowing or preventing the evolution of strong immune resistance, NAT maintains efficacy.

Cancer biologists have long assumed that enhancement of the host immune system could be an effective treatment strategy. However, only recently have effective immunotherapies emerged. A number of immunotherapies are now available to successfully treat a wide range of cancers. However, like other cancer treatments, malignant cells usually evolve resistance [34,35], leading to cancer progression. We hypothesize that the evolutionary principles that resulted in NAT could be actively used to both guide tumor prevention strategies and increase the efficacy of immunotherapy.

## Possible evidences for NAT

We assert that if NAT is mechanistically possible, then it should have been favored by natural selection and present in both humans and animals. The immune system and its function holds promise as a source of NAT. One of the most intriguing aspects in the relationship between the immune system and oncogenic processes is that the immune system does not always eliminate malignant cells [36,37] and sometimes even protects tumors and/or favors tumor growth [12, 36, 38–42]. While this is currently considered a problem to be solved to improve immunotherapies, it is potentially an illustration of NAT and the Darwinian dynamics that govern tumor resistance.

Growing tumors acquire intrinsic properties to inhibit immune effectors as well as to develop a microenvironment that prevents attack by diverse immune cells from the two arms of immunity, a phenomenon often referred to as immune escape. A number of mechanisms to escape acquired immunity have been identified. First, tumors can suppress immune response simply by generating a harsh environment. Disordered angiogenesis and chaotic blood flow can cause hypoxia and acidosis, which inhibits both vascular delivery of immune cells and reduces the antitumor activities of cytotoxic T lymphocytes [43]. Intrinsic properties of the tumor cells can decrease antigen presentation to the immune system by decreasing expression of MHC class I expression proteins to escape recognition by CD8 T cells [34,35]. Cancer cells can also escape the innate immunity by over expressing protective signals like CD47 to avoid antitumor activity of macrophages [44] and ADAM10 [43] to inhibit the antitumor activity of natural killer (NK) cells.

There is also a striking analogy between the tumor–host dynamics and the composition of wounded tissues undergoing repair [45]. For instance, wounded tissue is rapidly infiltrated with innate immune cells like neutrophils and macrophages that initiate and facilitate tissue repair. Likewise, this process is also associated with the formation of new blood vessels and the activation of fibroblasts into myofibroblasts that produce large amount of extracellular matrix proteins. Whereas these different cells and elements clearly restore tissue organization, in cancers, their persistence is associated with immune-modulating effects including the suppression of T-cell antitumor activities [46]. Similarly, there is often an excess of collagen fibers in growing tumors similar to that of healing wounds that can down-regulate the migration of T lymphocytes and their ability to contact and kill tumor cells [36,40].

In addition, it is now clearly demonstrated that some types of immune cells, beneficial in the context of tissue repair, can prevent other immune cells from eradicating cancer cells [42]. For example, regulatory T lymphocytes (Tregs) inhibit numerous antitumor activities of the immune system. Interestingly, increased numbers of Tregs associated with solid tumors has been associated with increased probability of both favorable and unfavorable outcomes in clinical studies [47,48]. Similarly, in wound healing, normal macrophages have distinct subtypes that either drive the early inflammatory responses (M1 type) or promote tissue repair (M2 type). Tumor-associated macrophages (termed TAM) resemble M2 type macrophages in wound healing and may promote tumor cell proliferation through a number of mechanisms, including angiogenesis and impairment of T cell-dependent antitumor immunity [38,39].

**Fig 3:**
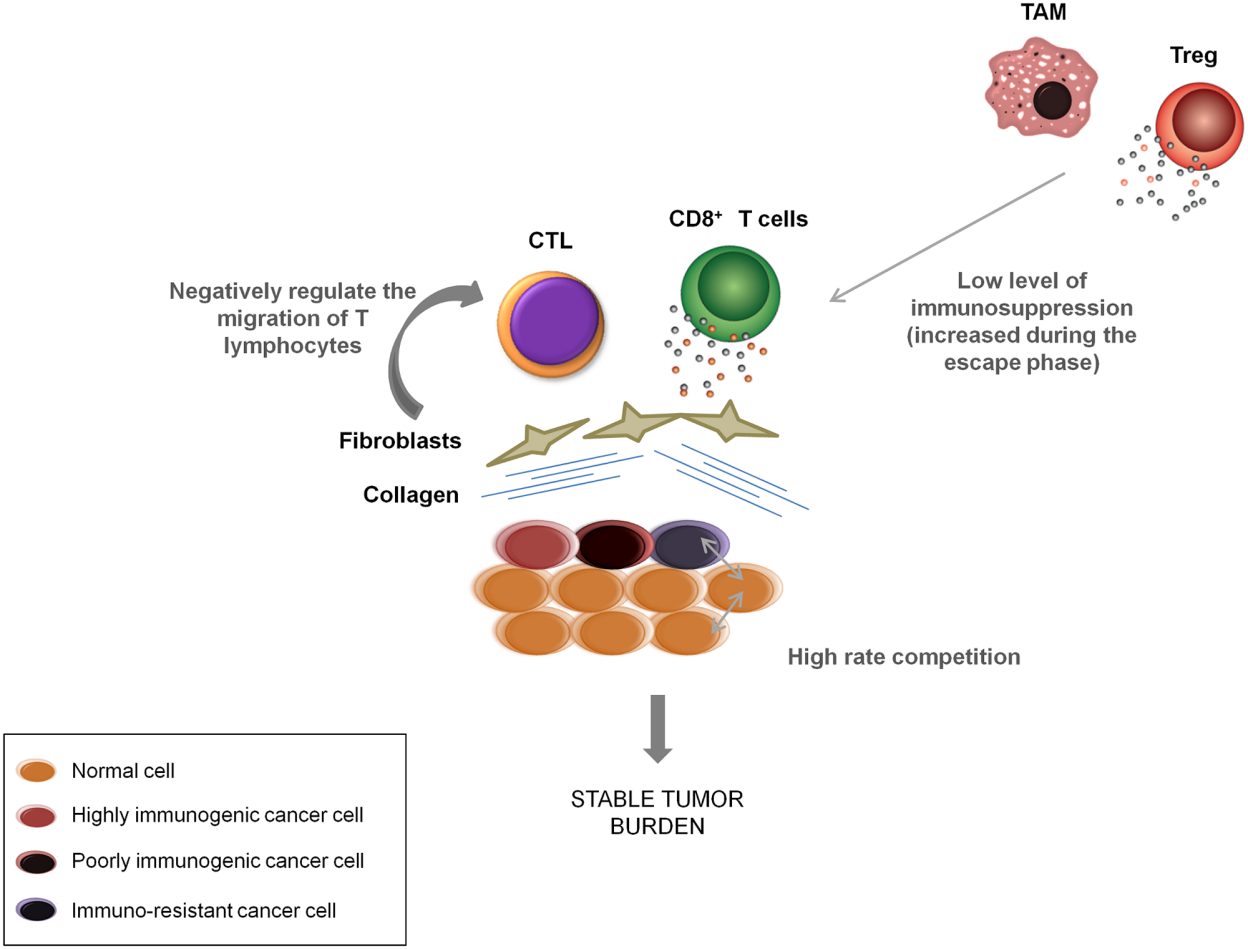
NAT hypothesis applied to the equilibrium phase of immunoediting. If the immune system cannot fully eliminate cancer cells, it could adopt NAT to limit impact on fitness through an equilibrium phase. During this phase, the immune system could maintain a stable tumor burden to increase intratumor competition and therefore delay the apparition of immuno-resistant cells. CTL, cytotoxic T cells; DC, dendritic cells; NAT, natural adaptive therapy; TAM, tumor-associated macrophages; Treg, regulatory T lymphocytes.

The evolutionary strategies that allow tumor cells to evade immune attack are perhaps best demonstrated in immunoediting. The three different phases of immunoediting are often characterized as the “three Es”: Elimination, Equilibrium, and Escape [49]. Within our NAT conceptual model, the equilibrium phase is particularly intriguing. This phase, which can occur over years to decades, may be evident in tumor dormancy, as small tumors remain undetectable for years before they eventually evolve into an aggressive disease [50]. The immune system may strongly influence or even regulate the dormancy/equilibrium state. Experimentally, tumor dormancy can be observed when cancer cells are grafted into immunocompetent mice. This dormancy can last for very long periods (hundreds of days). However, when grafted into immunocompromised mice, tumor growth and progression begins immediately [51]. The characterization of tumor dormancy remains poorly understood. It could result from cancer cell dormancy or, more likely, correspond to a stable tumor mass in which cancer cell proliferation and death balance, as defined in the equilibrium phase [52]. Even with a small and stable tumor mass, competition for space and resources could be intense within and between populations of cancer cell types such as those sensitive to and those resistant to the immune system (Fig 3).

## Therapeutic implications

Interfering with the adaptive response of the body carries some risk ([23]). For instance, reducing fever (which is an adaptation against infectious agents, notably bacteria [53]) is counterproductive if antibiotics are not concomitantly prescribed. If NAT exists and is adaptive, therapies that force the immune system to increase the kill rate of malignant cells may carry significant risk and could even backfire, see for instance [54,55]. This is clearly acceptable in immunotherapies directed against a life-threatening cancer. However, it seems likely that a full understanding of the strategies used by the immune system to contain but not eradicate tumors might allow therapies that are effective for longer time periods with reduced toxicity (e.g., [56,57]). Thus, tailoring of immunotherapies to complement the benefits of NAT would be an elegant and effective strategy (see also [58]). It appears also relevant in a therapeutic perspective to distinguish between individuals harboring rapidly progressing cancers and those with early stage tumors or premalignancies, since the latter may not benefit from immunotherapy. This is particularly important as trials are being developed that use checkpoint inhibitors like anti-PD1 as a cancer prevention strategy in individuals at high risk [59,60].

Alternatively, our hypothesis suggests strategies to prevent malignancies from evolving to disseminated lethal forms that might focus on maintaining NAT beyond the reproductive age. This preventive option would thus allow us to benefit longer from a natural defense mechanism; because this defense is “natural,” we can also speculate that it should be both easier (from a technical point of view) to (i) prolong a process that already exists and (ii) generate fewer side effects.

Finally, while we argue here that the immune system is the mediator of NAT, other tumor suppressive mechanisms may be reined in to achieve NAT. For example, it is possible that apoptotic responses to oncogenic pathway activations have been “tuned” to maximize individual fitness (i.e., balancing the tumor eliminating benefits of apoptosis with its costs, both in terms of increased selective pressures on cancer cells as well as costs to normal tissues). Polymorphism of the p53 locus at the R72 allele, which influences apoptotic responses to damage and which are differentially prevalent in humans in different parts of the world, may represent an example of competing costs [61]. Further research is also needed to evaluate the extent to which, in the context of NAT, malignant dynamics is influenced in immune-privileged organs like the brain, ovary, eye, and testis. Future studies will also need to elucidate the degree of NAT specificity, whether it is a specific anticancer adaptation and/or it emerges as a consequence of broader selective pressures on immune tuning.

Among the priorities for research that would establish or refute NAT, we suggest reinforcing strategies that would partially eliminate cancer cells in order to retain immune-sensitive tumor cells that would outcompete immune-resistant tumor cells, for instance, by targeting the tumor stroma instead of only targeting cancer cells. In progressing tumors, the stroma supports tumor growth by a variety of mechanisms, i.e., the “seeds and soil” hypothesis [62]. The goal would be to slow down tumor development by reducing the soil. Carcinoma-associated fibroblasts represent an interesting target, and several strategies have been developed [63]. Another direction would consist of decreasing the strength of the immune attack. Chimeric antigen receptor (CAR) T cells are used to eliminate cancer cells, with the drawback of killing normal cells expressing a tiny amount of the target. In addition, resistant tumor cells can emerge, which results in tumor relapse. An alternative approach would be to reduce the affinity of the CAR. There is preclinical evidence available for such a strategy with CAR T cells, the treatment having lower toxicity and, according to the NAT theory, more control over the tumor growth [64].

## Abbreviations

CAR: chimeric antigen receptor
NAT: natural adaptive therapy
NK: natural killer
TAM: tumor-associated macrophages
Treg: regulatory T lymphocyte
